# Predicted Distribution of Major Malaria Vectors Belonging to the *Anopheles dirus* Complex in Asia: Ecological Niche and Environmental Influences

**DOI:** 10.1371/journal.pone.0050475

**Published:** 2012-11-30

**Authors:** Valerie Obsomer, Pierre Defourny, Marc Coosemans

**Affiliations:** 1 Department of Parasitology, Prince Leopold Institute of Tropical Medicine, Antwerp, Belgium; 2 Earth and Life Institute Environmental Sciences Department, Université catholique de Louvain, Louvain-la-Neuve, Belgium; Instituto de Higiene e Medicina Tropical, Portugal

## Abstract

Methods derived from ecological niche modeling allow to define species distribution based on presence-only data. This is particularly useful to develop models from literature records such as available for the *Anopheles dirus* complex, a major group of malaria mosquito vectors in Asia. This research defines an innovative modeling design based on presence-only model and hierarchical framework to define the distribution of the complex and attempt to delineate sibling species distribution and environmental preferences. At coarse resolution, the potential distribution was defined using slow changing abiotic factors such as topography and climate representative for the timescale covered by literature records of the species. The distribution area was then refined in a second step using a mask of current suitable land cover. Distribution area and ecological niche were compared between species and environmental factors tested for relevance. Alternatively, extreme values at occurrence points were used to delimit environmental envelopes. The spatial distribution for the complex was broadly consistent with its known distribution and influencing factors included temperature and rainfall. If maps developed from environmental envelopes gave similar results to modeling when the number of sites was high, the results were less similar for species with low number of recorded presences. Using presence-only models and hierarchical framework this study not only predicts the distribution of a major malaria vector, but also improved ecological modeling analysis design and proposed final products better adapted to malaria control decision makers. The resulting maps can help prioritizing areas which need further investigation and help simulate distribution under changing conditions such as climate change or reforestation. The hierarchical framework results in two products one abiotic based model describes the potential maximal distribution and remains valid for decades and the other including a biotic mask easy to update with frequently available information gives current species distribution.

## Introduction

The *Anopheles dirus* complex (Peyton & Ramalingam, 1988) [1] includes the most efficient malaria vectors of Asia and species transmitting Artemisin-resistant malaria parasites which could compromise control efforts globally [2]. If the complex is typically associated with forests, specimens have been recorded in other landscapes reshaped by human activities such as orchards [3]–[6]. In countries where vast areas have not yet been surveyed, overall mapping is needed to allow targeting priority areas for additional surveys and surveillance. A geographical review gathering current ecological knowledge and environmental preferences for the seven species of the complex, and 200 geo-referenced collection sites including literature records proposes a general distribution map of known occurrences [7]. Similar geo-referenced sites have been used to predict the distribution of *An. dirus sensu lato* using presence-absence model where artificial absences are based on expert knowledge [8].

Various methods derived from the ecological niche concept [9] offer successful techniques to map species literature records [10]–[14]. Particularities of such datasets include low geographical precision and presence-only records. Indeed, site location is often not accurate and success in capture depends on sampling technique, seasonality and short-term changing meteorological conditions impeding the record of reliable absences. Additionally, remote sensing and Geographical Information Systems (GIS) technologies increase the availability of environmental digital datasets and geo-referenced species occurrence data which can be combined to estimate species distribution over a large region at coarse scale [15]. Amongst those new modeling techniques, the Maxent method selected for this study [13], [16] performs particularly well [10], does not require absence data and can be transferred to large areas with sparse or no species sampling records.

Additionally, the above method is adapted to integrate the hierarchical framework suggested by Soberon [17]. Indeed, explanatory factors used in models should be relevant for the timeframe and scale of the data. The distribution area is here defined in two steps. First slow changing abiotic factors are adapted for models based on historical records at coarse scale identify distribution areas according to physiological limits of the species (Grinnellian niche) [18] or what is considered here to be the potential niche. These factors include for *An. dirus s.l.* rain abundance and pattern, temperature, topography, soil type and relative humidity [7]. The second step help evaluate the realized niche (Eltonian niche) [19] defined by reducing the potential niche according to relevant biotic factors available at finer scale. Biotic factors, accounting for interactions with other organisms (including vegetation), change fast and must be studied using recent data. Current forest coverage is the most relevant biotic factor driving *An. dirus s.l*. distribution.

**Figure 1:**
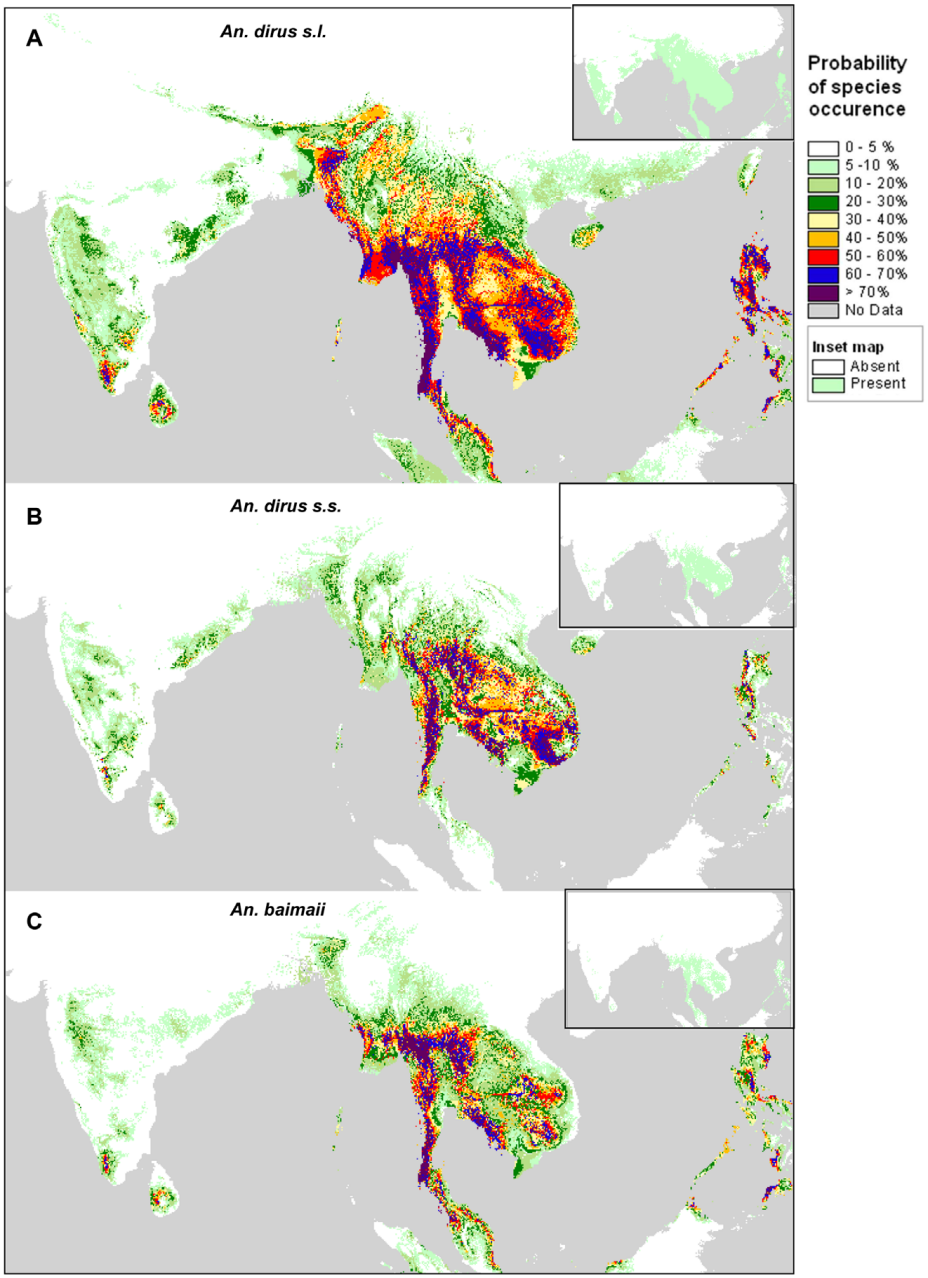
Probability of species occurrence. A) *Anopheles dirus sensu lato*, B) *Anopheles dirus sensu stricto* and C) *Anopheles baimaii*. Probability maps built using the mean of 100 replicates of the ECOOPT 1 km model based on 75% available samples. Inset maps present for each species presence/absence derived from the probability of species occurrence map based on 75% sample using as suitability threshold the value which maximizes sensitivity and specificity.

This study further tried to delineate distribution per species using the same methodology and to identify bionomics differences. Indeed, if the low number of sibling species records challenges potential analysis, clarifying bionomics differences and distribution per species is seen as a prerequisite for efficient control because of behaviour differences influencing efficiency of particular control measures [8].

The objectives of this study are to (1) Delimit the potential range of distribution for the Dirus complex (*An. dirus sensu lato*) and siblings species in Asia, (2) Assess the influence of environmental factors, (3) Identify potential differences between sibling species.

## Materials and Methods

The analysis was carried out in four steps illustrated in Figure S1. The two first steps mapped the “potential” and “current distribution” areas while step 3 compared distribution areas between the sibling species and step 4 analyzed the possible influence of abiotic environmental factors.

**Figure 2:**
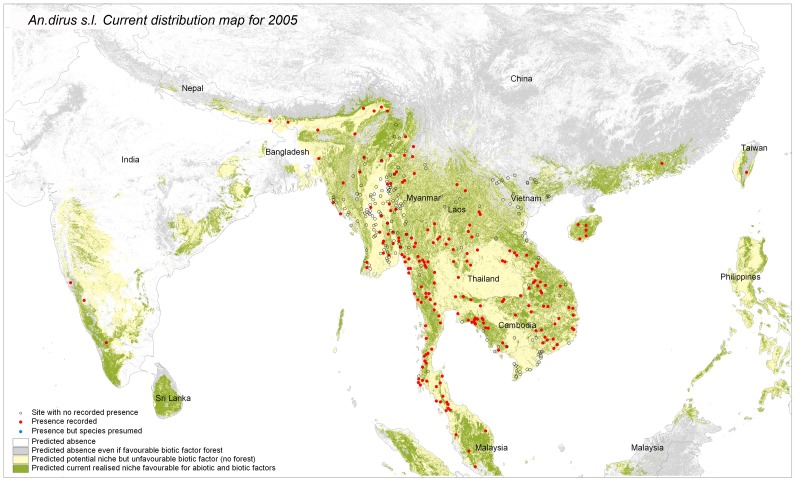
Predicted potential and current distribution area for *Anopheles dirus sensu lato.* The produced maps show in grey forested areas not suitable according to abiotic factor, in yellow the potential distribution based on abiotic factor but where forest is not present (potential niche) and the distribution as defined by favorable abiotic and biotic factors (“realized niche”). Performance tests for the model include test Gain (1.38), test AUC (0.90) and test extrinsic omission rate based on maximum test sensitivity plus specificity (6%).

### Entomological Data

The *An. dirus* complex belongs to the Leucosphyrus group (Reid, 1949) and occurs from India to Taiwan. *An. dirus* Peyton & Harrison, 1979 or *An. dirus sensu stricto*, *Anopheles crascens* Sallum & Peyton, 2005, *Anopheles scanloni* Sallum & Peyton, 2005 and *Anopheles baimaii baimaii* Sallum & Peyton, 2005, are recognized vectors of human malaria, while *Anopheles elegans* (James, 1903), *Anopheles nemophilous* Peyton & Ramalingam, 1988 and *Anopheles takasagoensis* Morishita, 1946 probably only transmit simian malaria [20]. The mosquito records were located in 370 sites including 199 positive sites for *An. dirus s.l.* Sites were prospected from 1974 to 2005. In some cases the species could be presumed from the location of the site. Available information included per species: *An. takasagoensis* (1 recorded), *An. dirus s.s.* (54 recorded +32 presumed), *An. crascens* (10 recorded), *An. scaloni* (8 recorded), *An. baimaii* (31 recorded +39 presumed), *An. elegans* (1 recorded +2 presumed), *An. nemophilous* (17 recorded). Two records were assigned to *An. dirus s.l.* because of unexpected identification of *An. dirus s.s.* and *An. scaloni* in Myanmar [21], [22]. Twenty three sympatric sites were recorded. Only presence records were used to develop the models. Presumed presences were just added for discussion to the results. *An. elegans* and *An. takasagoensis* were not studied due to low number of records. The complete dataset has previously been published [7].

**Figure 3:**
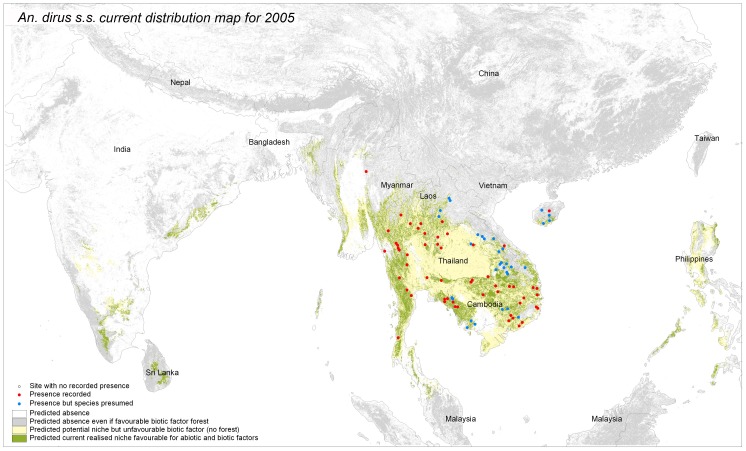
Predicted potential and current distribution area for *Anopheles dirus sensu strico*. The produced maps show in grey forested areas not suitable according to abiotic factor, in yellow the potential distribution based on abiotic factor but where forest is not present (potential niche) and the distribution as defined by favorable abiotic and biotic factors (“realized niche”). Performance tests for the model include test Gain (2.091), test AUC (0.9554) and test extrinsic omission rate based on maximum test sensitivity plus specificity (2.5%).

**Figure 4:**
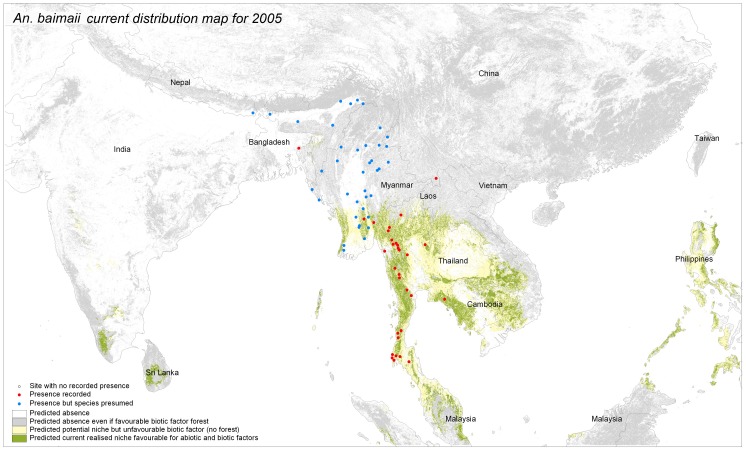
Predicted potential and current distribution area for *Anopheles baimaii.* The produced maps show in grey forested areas not suitable according to abiotic factor, in yellow the potential distribution based on abiotic factor but where forest is not present (potential niche) and the distribution as defined by favorable abiotic and biotic factors (“realized” niche). Performance tests for the model include test Gain (1.7), test AUC (0.93) and test extrinsic omission rate based on maximum test sensitivity plus specificity (5.7%).

**Figure 5:**
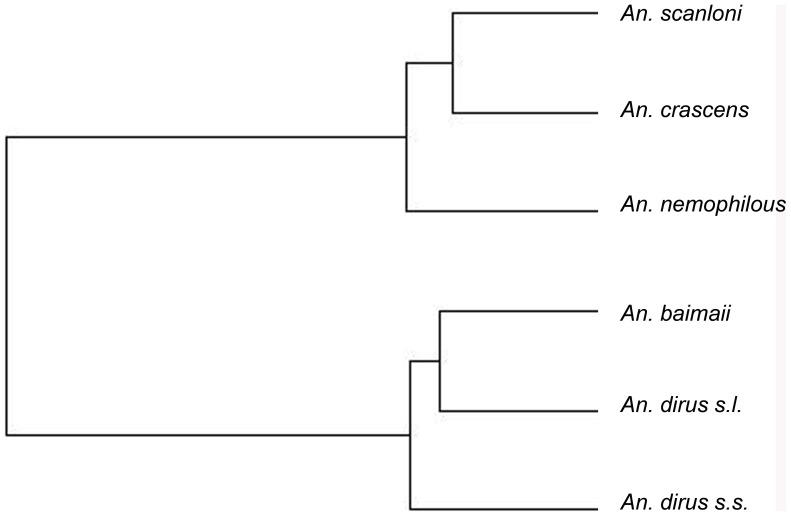
Similarity dendrogram. Similarity dendrogram based on Ward clustering method and modified Hellinger distance.

### Abiotic Environmental Data

Long term climatic datasets of monthly temperature and rainfall came from Worldclim [23] which provided also bioclimatic variables at 1 km, and CRU CL2.0 [24] which provided also number of monthly rainy days, rainfall monthly variation and relative humidity at 15 km resolution. The datasets are based on meteorological stations data from 1950 to 2000. The mean, median, maximum, minimum and standard deviation of the monthly values were calculated, as well as the number of months with mean temperature under 20 degrees and with less than 5 rainy days.

**Table 1:** Performance from univariate models.

	Variable description	Label	*An.dirus.s.l.*	*An.dirus s.s.*	*An. crascens*	*An. scanloni*	*An. baimaii*	*An. nemophilous*
A	Mean monthlyprecipitation	ANNRAIN	>1500 mm*	-	>2000 mm*	–	>1200 mm*	–
D	Rain Season (coeff.Variation)	ANNCVRAIN	–	65–95*	<65*	–	–	–
C	Lowest nber rainy daymonth	MINDAYRAIN	–	2–5 days*	–	–	1–4 days *	–
B	Highest nber rainy daymonth	MAXDAYRAIN	>20 days*	>20 days*	–	>20 days*	>21 days***	>20 days*
G	Nber month less5 rainy days	M5DAYRAIN	2–6 months*	1–5 months*	–	–	–	–
F	Nber months meantemp<20°c	M20MEANT	0 month*	0 month***	0 month***	0 month***	<3 months*	0 month**
H	Minimum tempwarmest month	MMAXMINT	19–25°C*	22–25°C*	–	–	22–25°C*	–
E	Maximum tempcoldest month	MMINMAXT	>24°C*	>26°C**	>28°C***	>28°C***	>28°C***	>26°C***
	Minimum tempcoldest month	MMINMINT	11–22.5°C*	12.5–21°C***	>20°C***	>15°C**	>12.5°C**	>14°C***
J	Mean of meanmonthly temp	MMEMEANT	23–27.5°C*	24–27.5°C*	>25°C**	>26°C**	24–27.5°C*	>20°C*
I	Std dev meanmonthly temp	MSTDMEANT	1–5°C***	0.5–2.5°C***	<1°C***	<2°C***	0.5–2.5°C***	<2.25°C***
L	Mean temp wettestquarter	WQMEANT	24–28°C*	25–27.5°C*	–	–	–	–
K	Mean temp driestquarter	DQMEANT	18–27°C*	22–26°C**	>25°C***	>24°C**	>22°C**	>22°C*
	Minimum relativehumidity	MMINREH	–	57–%*	>72%**	–	–	>67%*
	Minimum monthlywind speed	MMINWIND	–	–	–	–	1.25 m/s*	–
	Main soil groups	SOILS	–	acrisol**	fluvisol*	–	–	acrisol,fluvisol*
	Soil moisture storagecapacity	STORMAX	*	*	–	*	*	–
	Elevation abovesea level	DEM	–	–	<200 m*	–	–	–

Gain or performance from univariate models: – (<0.25),–(0.25–0.5), *(above 0.5), **(above 0.75) and ***(above 1). Variables with training gain under 0.5 for all species are not presented and include: MMAXRAIN, QMAXRAIN, NMINRAIN, QMINRAIN, MMINCVRAIN, MMENSUN, SLOPE, FLOW. For each relevant variable, the suitability value for presence is defined. The letters refers to the text and to Figure 4.

Soil types came from the Food and Agriculture Organization (FAO) soils maps (http://www.fao.org/ag/AGL/agll/dsmw.htm) including drainage, texture and salinity at 15 km resolution and updated version by the United State Department of Agriculture (USDA) at 4 km (http://soils.usda.gov/use/worldsoils/mapindex/order.html). Elevation, slope, flow direction, flow accumulation and compound topographic index came from the United State Geological Survey Gtopo30 (USGS) (http://edc.usgs.gov/products/elevation/gtopo30/hydro/index.html) at 1 km. All prepared sets were kept in their original resolution but cut to a same spatial extent corresponding to the Asia-pacific biomes [25] and known distribution range of *An.dirus s.l.* [7]. Data processing and calculation were performed using ArcGIS 9.3 [26] and ENVI 4.4 [27].

**Figure 6:**
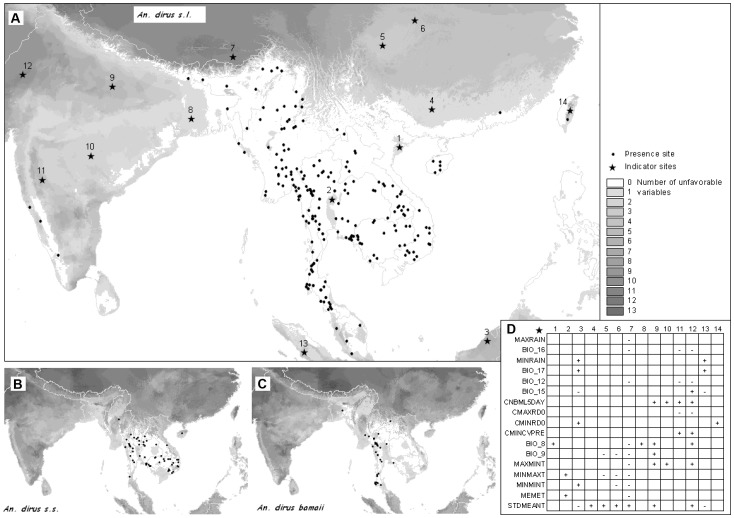
Environmental envelopes. A) The maps depict the maximum distribution range based on extreme limit values. A graded shade of grey depicts areas with unfavorable value for one or more variables, white being favorable areas for A) Dirus complex, B) *An. dirus s.s.* and C) *An. baimaii*. Some sites represented by a star symbol were selected in unfavorable areas. D) A small table analyze if the value is higher (+) or lower (−) to the actual range of value for each variables.

**Figure 7:**
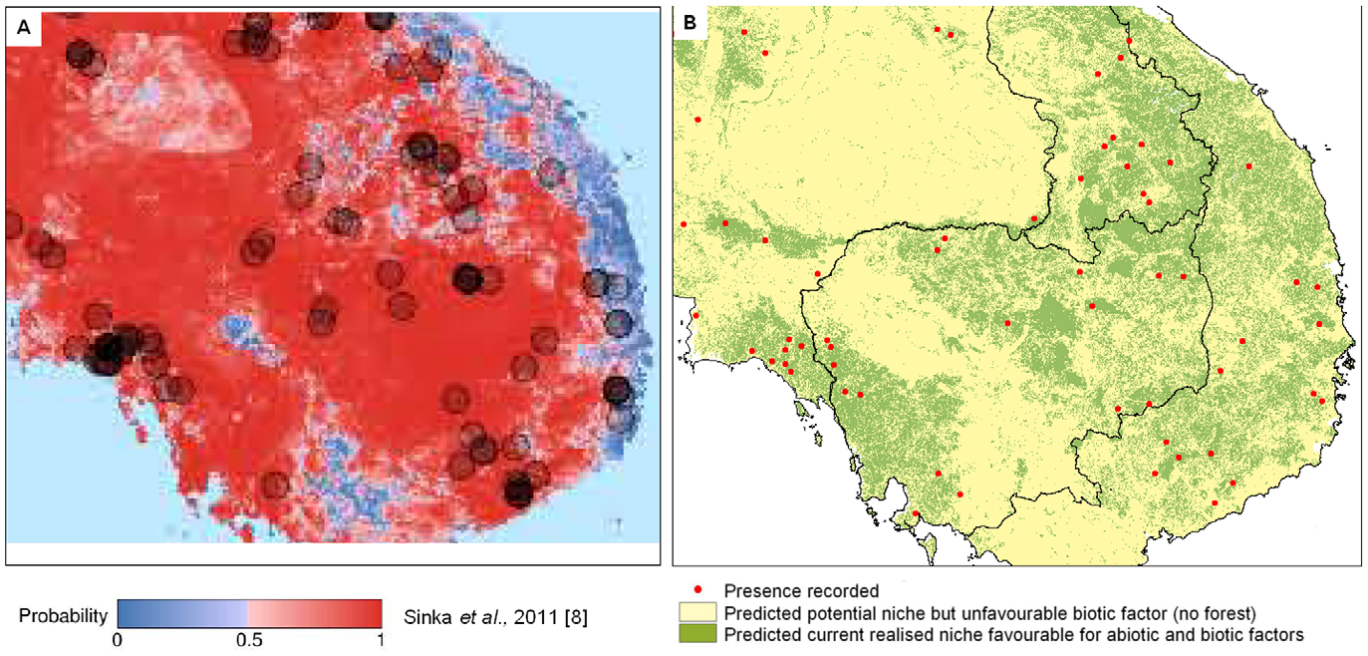
Comparison between *Anopheles dirus sensu lato* predicted distribution maps. Illustration of differences between the A) predicted distribution developed using Boosted Regression Tree presented by the Malaria Atlas Project [8] and B) the current distribution map developed in this paper.

### Biotic Data

Two forest masks were built to answer the needs of the current research.

The first mask provided the highest resolution available to build the most detailed distribution map. Forest habitat and land cover were provided for the world by the European Spatial Agency (ESA) Globcover product at a resolution of 300 m [28]. The classification was based on Meris satellite time series for year 2005 (http://ionia1.esrin.esa.int/) further processed in seasonal and annual composites. The land cover was reclassified into a binary forest/non forest cover to create a forest mask at 300 m resolution including classes: broadleaved and needle-leaved, evergreen or deciduous forest and woodlands a well as mosaic of forest with croplands and grasslands.

Calculation of niche similarity metric required masking the model with a 1 km resolution mask. To build this mask, the Greenness of vegetation index (NDVI) and wetness index (NDWI) were derived from spot VEGETATION satellite yearly composite images for 2005. NDVI and NDWI layers were calculated using ENVI software [27] and are based on annual composites of daily SPOT VEGETATION images prepared using the mean compositing method [29]. Grid cells with NDVI value below 0.5 and NDWI under 0.3 were classified as non forest.

### Step 1: The Fundamental Niche

#### Data reduction and groups of variables

To choose between the hundred abiotic variables at 1 km and 15 km, the variables were classified according to their possible contribution to very basic ecological questions: is there enough or too much rain? Is the rain and temperature pattern adequate? Is the temperature too high or too low? Are soils and topography adapted to breeding sites? Following Buermann [30], the covariance between pairs of variables was estimated. Single variable Maxent models [13] assessed importance of each variable to the distribution of *An. dirus s.l.* and trends of observed frequency and predicted suitability curves were compared and check for coherence [30] in order to select which layers from correlated pairs (with Pearson’ correlations coefficient equal or higher than 0.8) to retain for further analysis. While Maxent deals elegantly with highly correlated variables without influence on the output, the selection of non correlated variables make biological interpretation easier. Based on those preliminary results, one or two variables were selected per ecological questions for the 15 km and the 1 km dataset.

Three groups of abiotic variables were evaluated (See Table S1): (1) the most important climate datasets regarding species biology (CLIM) (2) datasets corresponding to ecological questions (ECO) and (3) the 10 variables performing the best (ECOOPT). The models were built for the *An. dirus* complex, then for the sibling species *An. dirus s.s*., *An. baimaii*, *An. scanloni*, *An. nemophilous* and *An. crascens*. This led to a total of 36 models with 6 “species” modeled at 2 resolutions (1 and 15 km) with 3 groups of variables.

#### Modeling techniques

Following the ecological niche modeling concept, the model was determined from a set of gridded variables with information available for all grid cells in a study area. The methods first defined the suitable niche for a species from the environmental values in the cells of occurrence and then, identified other cells in the study area corresponding to similar environmental conditions. The selected method in this study is Maxent (version 2.1.28; http://www.cs.princeton.edu/~schapire/maxent/) which assessed the probability distribution of a species by estimating the probability distribution of the maximum entropy [13]. The algorithm performed iterations in which the weights associated with the environmental variables, were adjusted to maximize the average probability of the sampled point locations (or average sample likelihood), expressed as the training gain [13]. For all models run in this study, we used the Maxent default settings. The default output map interpreted as a relative suitability or probability for species occurrence is further transformed in percent of suitability. Transformation to a binary presence/absence map required selection of a threshold value. Four potential threshold were calculated to maximize different criterion: (1) equal test sensitivity and specificity, (2) maximum test sensitivity plus specificity, (3) balance training omission, predicted area and threshold value, (4) equate entropy of threshold and non-threshold distribution. The best performing threshold was selected as explained below [13].

#### Models evaluation

To challenge the models, the recommended 50% of the samples [31] were set aside for validation. The models were run 100 times with test samples chosen randomly at each repetition. As the choice of the threshold value between presence and absence could influence the quality of the result, we used threshold-dependent and threshold-independent evaluation methods.

Threshold-dependent evaluation based on presence/absence map relies on test statistics to evaluate if the model performs better than random [13]. In particular, the extrinsic omission rate, defined as the fraction of test localities that fall into pixels outside the predicted area, was assessed and a one-tailed binomial test determine whether the model predicts better than random. The lowest extrinsic omission rate also characterized the best model. Threshold independent evaluation was performed using value of the Area Under the ROC (Receiver Operating Characteristic) Curve (AUC) frequently used in species distribution modeling literature [32]. In this case, the AUC was typically used as a measure of model performance between models of the same species, which avoid the drawback recently highlighted in the use of this index [33]. The likelihood of the test point localities, or test gain, was also monitored.

#### Map building

The final model with the highest number of best performance indicators was run again with the same method but 25% of samples set aside in order to maximize information extracted from samples while still allowing testing of the model and definition of a threshold between presence and absence. The average of the 100 models was then mapped. To improve model accuracy for species with low number of samples the target-group background method was used [34].

### Step 2: Biotic Factors and “Pseudo Realized Niche”

The potential distribution maps derived from the models were based only on abiotic factors. In a second step (Dataset S1), the area defined as the potential niche by the best abiotic model was further refined using the forest/non forest mask at 300 m based on current vegetation status in the region. This will allow to evaluate a “pseudo realized niche” as many others factors are probably interacting to define the true realized niche.

### Step 3: Species Niche Similarities

To determine if species niches were significantly different, Warren *et al.* [34] recently proposed a new similarity metric which carries no biological assumption and is thus better adapted to ecological niche modeling than more conventional measures of niche overlap [35]–[36]. The metric is based on Hellinger distance [37] previously used to compare community composition across sites [38] and developed for comparing probability distributions. In our case, the metric was based on the difference between probability of occurrence for two species in a given grid cell integrated over the whole study area and transformed to get values ranging from 0 (no overlap) to 1 (identical niche). The metrics were calculated using ArcGIS over the “realized niche” derived from the best performing abiotic model and the forest/non forest mask. A similarity dendrogram was then built using the Ward’s minimum variance criterion.

### Step 4: Influence of Abiotic Environmental Factors and Related Envelopes

If causality cannot be derived from correlative analysis, the apparent correlation between species and environmental factors, including specific ecological limits which define environmental envelopes, can however be highlighted. In step 4 (Figure S1), the relative importance of each contributing factors to the model was first assessed by running a model using only one variable to predict the presence of each species and estimating the gain (model predictive power) associated to that variable. From each of the univariate suitability model with good performance (gain >0.75), a binary presence/absence map for the species considered was developed based on a threshold value which maximize sensitivity and specificity. The response curve from each univariate model was plotted with the concordant species sample frequency to infer ecological conditions relevant for the species (Figure S2) [30]. Threshold values were then derived if relevant for any species.

## Results

According to threshold dependent and independent evaluation methods (see Table S2), ECOOPT at a resolution of 1 km (ECOOPT1) was the best performing abiotic model. Performances were generally stable with highly performance for AUC values test (90%) and highly significant extrinsic omission rates of 50% test samples regardless of the criteria used to cut off suitability value between absence and presence. According to the threshold criteria (See Table S3), the suitability value selected as cut off between presence and absence varied according to the species. For all species, the threshold values selected following the criteria of maximizing the specificity and sensitivity offered the best discrimination with the highest cut off value for a low omission rate (1% or less) and was used for delineating subsequent potential distribution maps. The models developed for species with a low number of samples, *An. crascens*, *An. scanloni* and *An. nemophilous*, are less reliable due to the low number of samples compared to the number of variables.

### Step 1: Potential Distribution Area: the “Potential Niche”

The suitability maps based on the mean of the 100 replicates of model ECOOPT1 depict the potential distribution areas for the complex and the species with reasonable sample size (Figure 1). In each map a subset map represents presence/absence binary maps. The model based only on abiotic factors depicted properly the northern limit of the distribution of *An. dirus s.l*., absence in most parts of India, presence in the island of Hainan and absence in the north of Vietnam as observed in the field. The binary map of presence/absence was also consistent with known distribution. The potential distribution in Sri Lanka and Indonesia fitted with the occurrence of species belonging to other species of the Leucospyrus group. For species such as *An. dirus* s.s and *An. baimaii* with reasonable number of samples, distribution area was restricted compared to *An. dirus* complex and might be smaller than its actual distribution. This is particularly the case when looking at the binary presence/absence maps in the case of *An. baimaii* and there might be an artifact link to the choice of the threshold. When looking at the suitability maps instead of binary maps (Inset map Fig. 1), *An. baimaii* was predicted more southwards than its known distribution but was absent from the coast of Vietnam.

### Step 2: Current Distribution: “Realized Niche” Visual Interpretation

The potential species distribution was refined with the Globcover forest mask. *An. dirus s.l.* distribution was concordant with the known distribution, except from over prediction in Sri Lanka, The Philippines and Indonesia (Figure 2). If *An. dirus s.l*. had not been recorded there, other members of the Leucosphyrus group, which include the Dirus complex, are vectors in the area. This is the case at least for *An. mirans* in Sri Lanka, *An. baisasi* in the Philippines and *An. latens* in Indonesia [1], [39]. The forest mask eliminated central Thailand, parts of Cambodia and some areas of India. *An. dirus s.s.* distribution (Figure 3) fitted with known distribution apart from one site in central Myanmar. The species status for that site was however not confirmed [21]. The possible presence sites were few in Hainan Island. For *An. baimaii* (Figure 4), the area partly corresponded to known distribution for the species and the complex but the species was predicted to occur in south of India, although never reported there, in Cambodia although only *An. dirus s.s*. had been reported there, and under predicted in Myanmar.

### Step3: Niche Similarity

The ecological niche of *An. dirus* complex, *An. dirus s.s.* and *An. baimaii* were more similar (>0.75). *An. dirus s.s.* was closer to *An. baimaii*. *An. crascens* presented the ecological niche most at the margin of the complex and was very close to *An. scanloni* and close to *An. nemophilous*. (Figure 5).

### Step 4: Influence of Environmental Factors

The relative importance of each separated environmental variable as predictor of the distributions was estimated through the gain or prediction performance achieved creating univariate model based on this factor. The environmental variables which show good performance for at least one species are presented in Table 1 with their limit values. Temperature related factors had the highest influence on the distribution of the species. The uppercase letters in the following paragraphs refer to the Table 1 and Figure S4.

The annual rainfall (A: >1500 mm) and the rainfall distribution pattern with frequent rains in the rainy season (B: >20 days/month) and sporadic rains in the dry season (C) characterized the occurrence area for the complex, but the influence of rainfall was not very strong (gain <0.5) compared to temperature. *An. baimaii* occurred in areas presenting a lower amount of rainfall (A) but more frequent rain (B) while the distribution area of *An. crascens* was correlated with higher amount of rainfall (A). Regarding rainfall patterns, *An. dirus s.s.* distribution area was characterized by a shorter dry season (C) and a less regular annual pattern (D) than observed for *An. crascens*.

A mean temperature (F) of at least 20°C throughout the year seemed to characterize the distribution area of the complex with a mean annual temperature (J) ranging from 23–27.5°C and a variation of 1–5°C(I). Low temperatures seemed to limit the distribution to the north and high temperatures in west India, central Vietnam and central Thailand. Daily extreme temperature showed similar trends with maximum temperature never being too high and above 24°C during the coldest month (E). Minimum temperature was between 11–22.5°C during the coldest month and between 19–25°C during the warmest month (H). If studying interaction between rainfall and temperature, distribution areas presented a mean temperature of 24–28°C during the rainy season (L) and 18–27°C during the dry season (K). Species specific thresholds were overall similar with lower tolerance for temperature variation in general. *An. crascens* was found in areas with the smallest mean temperature variation (I <1°C) and the highest minimum (Figure S4) and maximum temperature (E).

The other variables were more difficult to interpret. The complex occurred mostly in area of low soil water storage capacity in Southeast Asia but also in high water storage capacity in India thus no clear trend could be derived from this variable. Associated soils included mainly ultisols which cover most of Southeast Asia. The minimum relative humidity ranged from 57–72% for *An. dirus s.s.* distribution area, above 67% for *An. nemophilous* and above 72% for *An. crascens*.

### The Environmental Envelope Approach

While limit values can characterize the behavior of outliers and thus not be representative of the general species distribution, the environmental envelope approach predicts the extremes in the distribution of species, which is useful to explain the incapability of species to colonize some areas (e.g. *An. dirus* in northern Vietnam). The map in Figure 6 showed in white the areas of suitable abiotic environment. Fourteen sites were selected on the map in regions predicted as unfavorable to the vector in order to investigate which variables presented values above or below the limit for those areas. For example when searching for a limit to the west (star 12) the annual rainfall as well as rainfall during the wettest quarter was low with a number of rainy day always low and too many months with less than 5 days rain. Temperature was high during the wettest quarter and minimum temperature was also high with high temperature variations. In eastern India (star 8), the situation was similar to the red river delta on the coast of Vietnam (star 1) with high mean temperature during the wettest quarter.

## Discussion

Using presence-only models and hierarchical framework this study managed not only to predict the distribution of a major malaria vector, but also improve ecological modeling analysis design and proposed final products better adapted to malaria control decision makers.

Lack of information on vectors distribution and environmental preferences are major drawbacks in malaria elimination effort [41]. With the paper Obsomer et al 2007, the current article collect most known location for the dirus complex, gather most of the knowledge on species ecology, produce maps per sibling species and propose potential and current distribution map for the complex. In addition to maps plotting occurrences, distribution models predict areas where the presence or absence is unknown such as South Nepal where few investigations have been carried out. In countries where vast areas have not yet been surveyed, it helps prioritize highest occurrence risk areas. Predictions based on environmental variables help simulate distribution under changing conditions such as climate change or reforestation. But moreover, the hierarchical framework improved the potential use of the results in several ways by (1) providing a potential distribution map based on abiotic factors and valid for several decades (2) providing a current distribution map based on vegetation coverage whose validity date can be identified, here the acquisition time of satellite images used to derive the biotic factors (year 2005) (3) providing easy update by non specialist. Indeed, regular update of mask of the fast changing biotic factors does not require rerunning the abiotic model and take advantages of increasingly available update of these products (4) avoiding the use of expert knowledge which make modeling a lengthy and costly process (5) providing GIS maps at 1 km for zooming in areas of interest and overlaying other layers (See Zip File Archive SGISFILE01) (6) providing suitability map ranging from 0–100% giving an estimated risk when decision makers decided to carry or not surveillance in a given area (7) providing two products for decision tool. Indeed, the potential distribution map of areas with favourable abiotic factors represents the maximal extent of the complex in case of reforestation. Thus favourable areas with forest delineate the current distribution area while favourable areas without forest are potential extension zones in case of reforestation. Such large scale reforestation is current in China and other countries of Asia [42] but newly available habitat might not be as suitable as natural forest. There the species might adapt to new biotic factors such as orchards [43]. Outside of this area, the abiotic factors are not favorable.

The method deals with two recurrent problems in ecology: unreliable absences and mapping old species data with fast changing biotic variables. Accurate distribution model were processed without requiring expert knowledge to correct or train the model. Expert knowledge can create bias in the analysis and quality of the information is difficult to assess or may vary regionally. The hierarchical framework improved the analysis design by modelling literature records based on variables relevant from the same time frame, while still integrating relevant biotic data. The separation of abiotic and biotic factors is not new [44]–[46]. If biotic factors such as forest cover are dependent of abiotic factors [47], current forest distribution is also the reflection of land use and thus a fast changing biotic factor. The use of biotic factor in a mask and not in the model avoided correlation issues [48].

*An. dirus s.l*. predicted distribution is consistent with its known distribution [7]. The models predict however presence of the complex in region where it has never been recorded such as in the Andaman Islands, the Philippines and Indonesia which are inhabited by related species of the Leucosphyrus group [49]. Those islands might not be accessible to the specie or alternatively, the genetic variation between the members of the Leucosphyrus group might not be linked to environmental preference but depends on evolution history [50]. The distribution maps for the sibling species reflect only partly the known distribution. Our study managed to highlight differences between species. It is however unknown if those are environmental preferences or species are developing at the margin of the complex distribution range in a less favorable environment. *An. crascens* and *An. takasagoensis* which live in the wettest areas are also the less efficient vectors. One could suggest that a given species is a vector in a source population and non vector if in a sink population. This is anyway not the case for *An. dirus s.s.* which transmits systematically the disease when present even if in areas favorable only in the rainy season. Rascalou (2012) also indicates that sink vector populations can represent serious threats to human health [51].

The distribution of *An. dirus s.l.* has been previously predicted by [8] using Boosted Regression Tree [10]. While this method produced valuable map for *An. dirus s.l*., it relies on absence data artificially created based on expert knowledge thus adding a bias unnecessary in our modeling technique. In this context we used a method proposed by Phillips [34] for selection of background data by including in pseudo absences a spatial bias similar to the potential bias of presence data. Species with few sampling sites were mostly discovered while searching for other more widely distributed species and probably present stronger sampling bias. Additional improvements are illustrated with Figure 7. (1) The use of higher resolution dataset allowed better delineation of favourable areas and reduced the superficies of the region requiring surveillance. While the resolution of the abiotic dataset used for modeling the potential distribution cannot be easily improved because of necessary adequacy with the precision of the site sampling location, the fact that the biotic factors are added as a mask allow to keep this dataset in full resolution and thus provide a more detailed resulting map (2) Strong deforestation occurs in Cambodia and a model integrating biotic factors averaged on 20 year period lose the pertinence of up-to-date information. The use of up to date biotic information for our model managed to capture this fast changes in the forest cover leading to a far more accurate map (3) The use of averaged biotic data in the model do not allow to give a precise date at which the model could predict valid distribution. Such model is thus difficult to use in the field. Our model is valid for year 2005. (4) Additionally, the output format in pdf does not allow zooming in area of interest and do not allow overlaying other layers. A GIS format allows better interaction with potential user and will lead to improved quality.

The models result is limited by the quality of the variables which present two main drawbacks. Ecological model should be based on source populations. Those are sustainable populations living in suitable habitat while sink populations are surviving in habitat not suitable for population persistence thanks to immigration from nearby source population. Typical museum records include both sink and source populations [17]. Entomological data are also mostly available from the nineties with the development of sibling species identification methods [1] while the climatic variables range from 1950 to 2000. Predictive mapping of species with low number of recorded occurrence could be improved by selection of a fixed threshold which was shown to improve model performance [52].

This study is a first step in delineating potential and current distribution of the *An. dirus* complex. It shows that using wide scale abiotic variables based mostly on climate, it is already possible to refine the potential distribution area. Fine scale mapping of other biotic factors relevant for the mosquito survival such as presence of host, breeding sites, state of the forest cover is needed to further reduce the predicted area. Those maps could thus be integrated into more functional models such as agent-based models [53]. Such models can be very useful for decision makers by providing a risk assessment to their mosquito control surveillance program and strategy. More detailed analysis using concomitant entomological data and biotic information could help to refine the distribution and favorable vegetation types. *An. dirus s.l.* is believed to recede during the dry season in forest areas where the moisture remains high [7]. Studies at highest resolution could localize the restricted area of distribution in the dry season to help in focus vector control activities.

In the context of climate changes, the land cover has been identified as one of the key variables influencing the climate (Essential Climate Variables) and will thus probably soon beneficiate of regular, standardized and faster updating of detailed information. Recently, major mapping effort for malaria vectors led to the gathering of large databases of record sites [8] which will offer more opportunity to test the methodology and refine the distribution.

## Supporting Information

Dataset S1 **Zip File Archive GIS files of the resulting map for** ***An. dirus sensu lato.*** GIS TIF image for visualization in ARCGIS software including the abiotic model result (dirus01.tif, 0: not favourable, 1: favourable), the forest mask (gcoforest01, not favourable, 1: favourable) and combined information (finaldirus, 0: not favourable, 1: abiotic favourable but no forest, 10: forested area but abiotic factors not favourable, 11: current distribution 2005.
(ZIP)

Figure S1 **General analysis scheme.** (1) Prediction of the fundamental niche based on abiotic factors, (2) Refining distribution to the “realized niche” based on biotic factors, (3) analysis of niche similarities (4) Correlation with the environment parameters and environmental limits.
(TIF)

Figure S2 **Example predicting the distribution of species using one of the abiotic environmental variables: minimal minimum monthly temperature – MINMINT.** A) The main map presents the environmental variable overlaid with presence absence information for *An. dirus s.l.* The small maps present the distribution area by species such as defined by univariate models developed using only that environmental variable with B) *An. dirus s.l.* E) *An. crascens*, C) *An. baimaii,* D) *An. dirus s.s.,* F) *An. scanloni,* G) *An. nemophilous*. H) A graph represents the MaxEnt response curves (lines) and sample density histogram (diamond) for *An. dirus s.l.* The response curve illustrates the predicted suitability for the species using that single environmental variable.
(TIF)

Figure S3 **Distribution maps for species with low number of samples.** A) *An. crascens*, B) *An. scanloni*, C) *An. nemophilous*. 1)Probability of species occurrence build using the ECOOPT 1 km model based on 75% available samples and accounting for sampling bias, 2)Presence/absence maps derived from the probability of species occurrence map based on 50% sample using as suitability threshold the value which maximize sensitivity and specificity, 3)Predicted potential and current distribution area. (grey: forested areas not suitable according to abiotic factor; yellow: potential distribution based on abiotic factor but where forest is not present (potential niche); green: the distribution as defined by favorable abiotic and biotic factors (“realized” niche). Performance tests for the model have similar values for the three species model include test Gain (1.42 to 2.04), test AUC (0.91–0.95) and test extrinsic omission rate based on maximum test sensitivity plus specificity (0% for the three species).
(TIF)

Figure S4 **Environmental influences.** Environmental factors correlated to the distribution area of the Dirus complex according to the results available in table 1. A) ANNRAIN: Mean monthly precipitation, B) MAXDAYRAIN: Highest number rainy day month, C) MINDAYRAIN: Lowest nber rainy day month, D) ANNCVRAIN: Rain Season (coefficient of Variation), E) MMINMAXT: Maximum temp coldest month F) M20MEANT: Number months mean temp<20°c, G) M5DAYRAIN: Number month less 5 rainy days, H) MMAXMINT: Minimum temp warmest month, I) MSTDMEANT: Std dev mean monthly temp, J) MMEMEANT: Mean of mean monthly temp, K) DQMEANT: Mean temp driest quarter, L)WQMEANT: Mean temp wettest quarter.
(TIF)

Table S1 **Abiotic environmental variables.** Selected variable for the 15 km and 1 km resolution niche models: Variable description, Ecological question (biological justification for selection of variables based on expert knowledge) Label (variable short name), presence in analysis at 1 km (1CLIM - climatic, 1ECO – all, 1ECOOPT – best) and 15 km resolution (15CLIM, 15ECO, 15ECOOPT).
(XLS)

Table S2 **Measure of model performances.** Threshold independent and dependent measures of model performance for three groups of variables (CLIM, ECO and ECOOPT), two resolutions (1 km, 15 km) using 50% of samples for testing. The best performance per test is in bold.
(XLS)

Table S3 **Cut off values to transform probability maps into binary maps for various threshold indices.** Cut off values used to transform probability maps into presence/absence maps according to various threshold indices: V =  Cut off probability value, T = Test extrinsic omission rate, Equal spe&se = Equal test sensitivity and specificity, Max spe&se = Maximum test specificity plus sensitivity, Balance TPT = Balance Training omission, predicted area and threshold value, Equate entropy = equate entropy of thresholded and non thresholded distribution. Test extrinsic omission rates calculated on the binary map measure model performance according to threshold indices for best performing group of variables 1ECOOPT developed using 50% of samples for testing.
(XLS)
