# Supplementary figures and images for: Predicted Distribution of Major Malaria Vectors Belonging to the Anopheles dirus Complex in Asia: Ecological Niche and Environmental Influences
Source: PLoS One. 2012 Nov 30;7(11):e50475. doi: 10.1371/journal.pone.0050475 (PMC3511573; doi:10.1371/journal.pone.0050475)

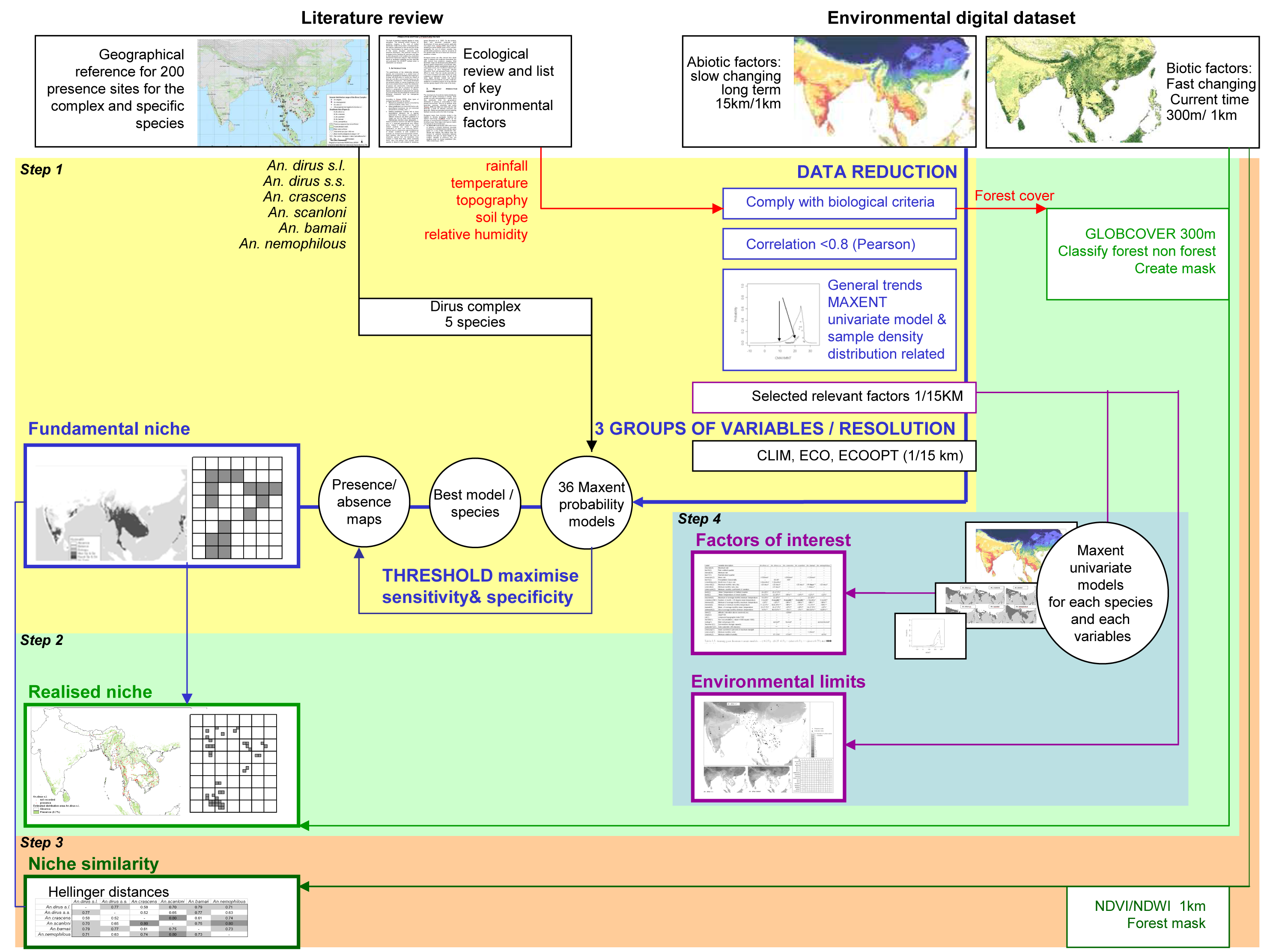

Supplement: Figure S1 — General analysis scheme. (1) Prediction of the fundamental niche based on abiotic factors, (2) Refining distribution to the “realized niche” based on biotic factors, (3) analysis of niche similarities (4) Correlation with the environment parameters and environmental limits. (TIF) [file pone.0050475.s002.tif]

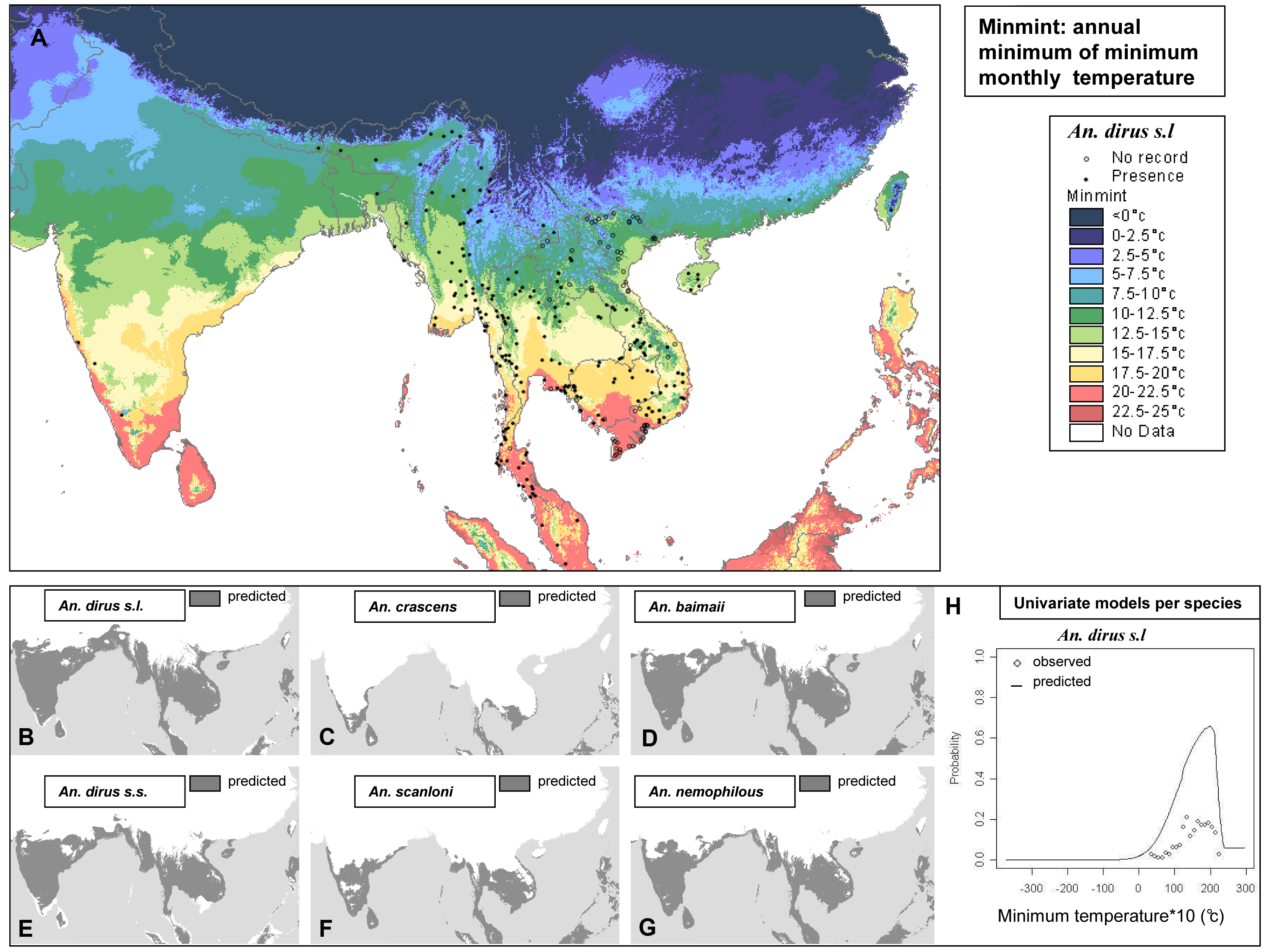

Supplement: Figure S2 — Example predicting the distribution of species using one of the abiotic environmental variables: minimal minimum monthly temperature – MINMINT. A) The main map presents the environmental variable overlaid with presence absence information for An. dirus s.l. The small maps present the distribution area by species such as defined by univariate models developed using only that environmental variable with B) An. dirus s.l. E) An. crascens, C) An. baimaii, D) An. dirus s.s., F) An. scanloni, G) An. nemophilous. H) A graph represents the MaxEnt response curves (lines) and sample density histogram (diamond) for An. dirus s.l. The response curve illustrates the predicted suitability for the species using that single environmental variable. (TIF) [file pone.0050475.s003.tif]

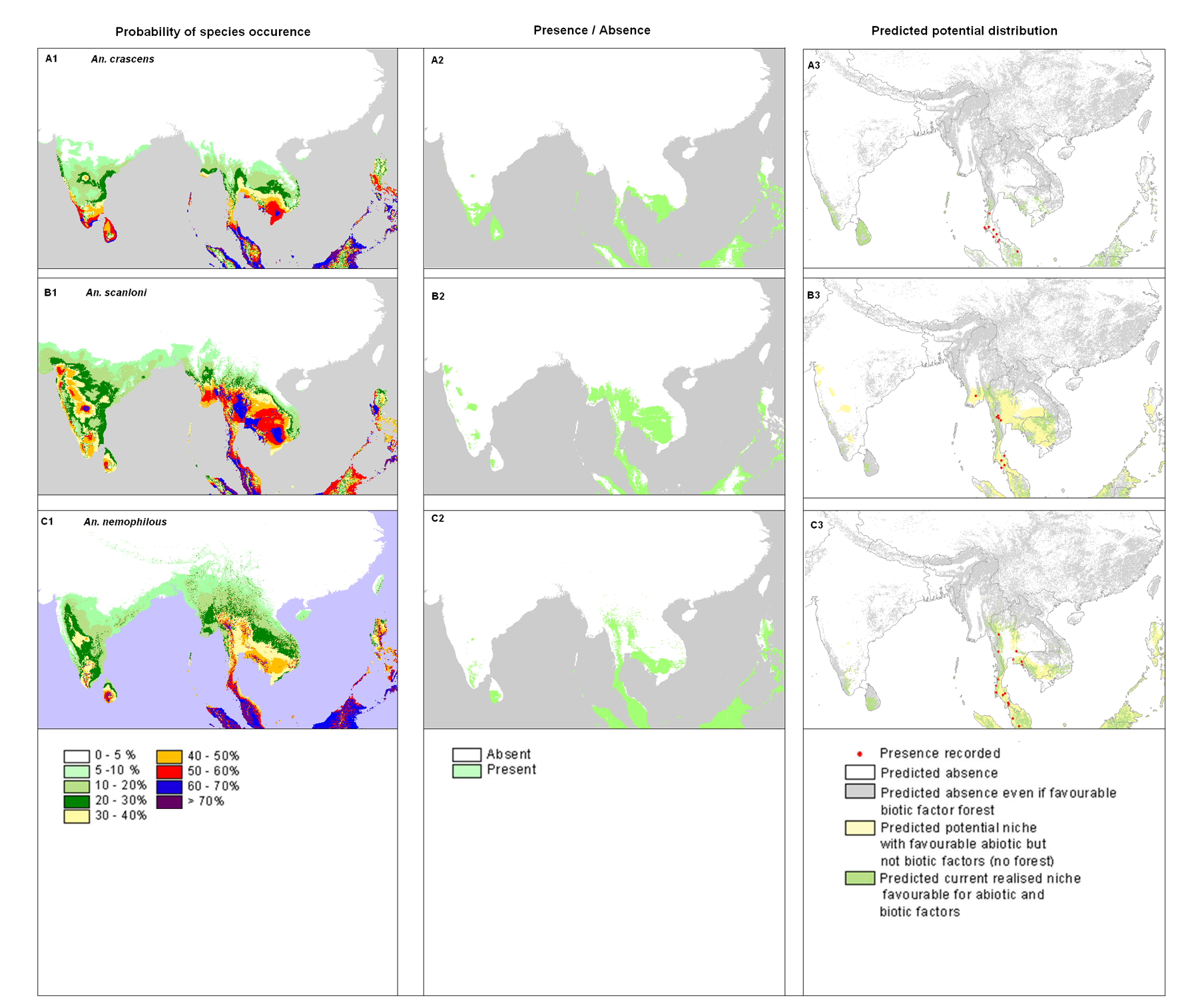

Supplement: Figure S3 — Distribution maps for species with low number of samples. A) An. crascens, B) An. scanloni, C) An. nemophilous. 1)Probability of species occurrence build using the ECOOPT 1 km model based on 75% available samples and accounting for sampling bias, 2)Presence/absence maps derived from the probability of species occurrence map based on 50% sample using as suitability threshold the value which maximize sensitivity and specificity, 3)Predicted potential and current distribution area. (grey: forested areas not suitable according to abiotic factor; yellow: potential distribution based on abiotic factor but where forest is not present (potential niche); green: the distribution as defined by favorable abiotic and biotic factors (“realized” niche). Performance tests for the model have similar values for the three species model include test Gain (1.42 to 2.04), test AUC (0.91–0.95) and test extrinsic omission rate based on maximum test sensitivity plus specificity (0% for the three species). (TIF) [file pone.0050475.s004.tif]

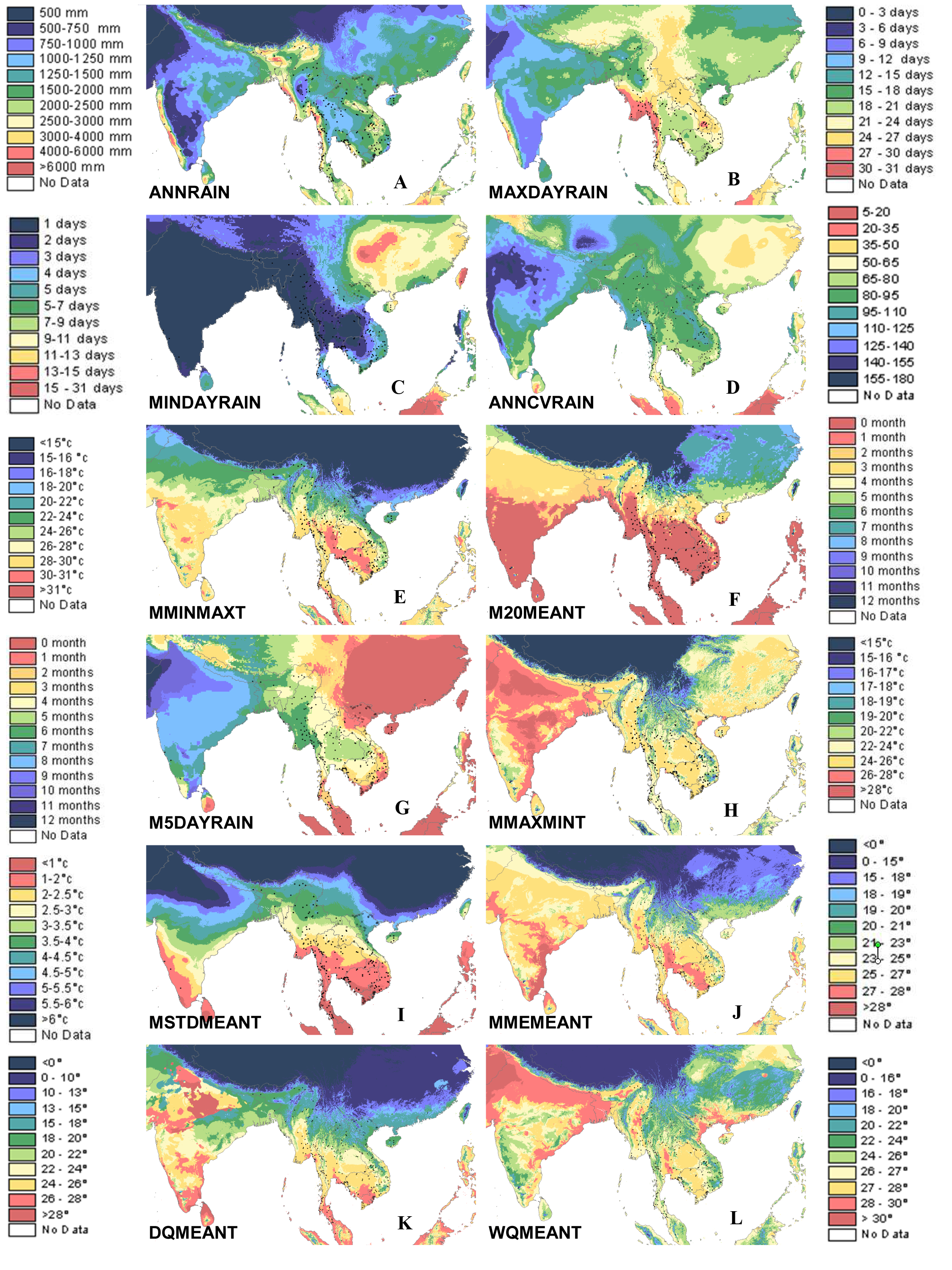

Supplement: Figure S4 — Environmental influences. Environmental factors correlated to the distribution area of the Dirus complex according to the results available in table 1. A) ANNRAIN: Mean monthly precipitation, B) MAXDAYRAIN: Highest number rainy day month, C) MINDAYRAIN: Lowest nber rainy day month, D) ANNCVRAIN: Rain Season (coefficient of Variation), E) MMINMAXT: Maximum temp coldest month F) M20MEANT: Number months mean temp<20°c, G) M5DAYRAIN: Number month less 5 rainy days, H) MMAXMINT: Minimum temp warmest month, I) MSTDMEANT: Std dev mean monthly temp, J) MMEMEANT: Mean of mean monthly temp, K) DQMEANT: Mean temp driest quarter, L)WQMEANT: Mean temp wettest quarter. (TIF) [file pone.0050475.s005.tif]
